# *Culicoides*-Specific Fitness Increase of Vesicular Stomatitis Virus in Insect-to-Insect Infections

**DOI:** 10.3390/insects15010034

**Published:** 2024-01-05

**Authors:** Paula Rozo-Lopez, Barbara S. Drolet

**Affiliations:** 1 Department of Microbiology, University of Tennessee, Knoxville, TN 37996, USA; 2 Arthropod-Borne Animal Diseases Research Unit, United States Department of Agriculture, Manhattan, KS 66502, USA

**Keywords:** vesicular stomatitis virus, VSV, maintenance, *Culicoides sonorensis*, biting midges, mammalian cell cultures, insect cell cultures, within-host fitness

## Abstract

**Simple Summary:**

Vesicular stomatitis virus (VSV) infects cows, horses, and pigs, economically impacting livestock producers due to animal production losses, quarantines, and animal movement/trade restrictions. Typically, VSV is transmitted from animal to animal by direct contact, but it is also transmitted by insects such as *Culicoides* biting midges. These tiny flies can ingest virus particles when blood feeding on an infected animal, multiply them inside their bodies, and then transmit them to other animals the next time they feed. In addition, midges are also able to pass the virus from one to another with extremely high efficiency when they mate, even though they carry very little virus in their bodies. Through this mechanism, VSV may be maintained and overwinter in midges and appear again in livestock the next summer once the insects start feeding on blood again. Our research shows that one reason midges can transmit VSV to other midges so efficiently is because viruses that come from insect cells have an increased ability to infect more insect cells. This helps explain the midge-to-midge infection efficiency and highlights the importance of *Culicoides* midges in VSV maintenance and transmission.

**Abstract:**

Vesicular stomatitis virus (VSV) is an arthropod-borne virus affecting livestock. In the United States, sporadic outbreaks result in significant economic losses. During epizootics, *Culicoides* biting midges are biological vectors and key to the geographic expansion of outbreaks. Additionally, *Culicoides* may play a role in VSV overwintering because females and males are capable of highly efficient venereal transmission, despite their relatively low virus titers. We hypothesized that VSV propagated within a midge has increased fitness for subsequent midge infections. To evaluate the potential host-specific fitness increase, we propagated three viral isolates of VSV in porcine skin fibroblasts and *Culicoides* cell lines. We then evaluated the viral infection dynamics of the different cell-source groups in *Culicoides sonorensis*. Our results indicate that both mammalian- and insect-derived VSV replicate well in midges inoculated via intrathoracic injection, thereby bypassing the midgut barriers. However, when the virus was required to infect and escape the midgut barrier to disseminate after oral acquisition, the insect-derived viruses had significantly higher titers, infection, and dissemination rates than mammalian-derived viruses. Our research suggests that VSV replication in *Culicoides* cells increases viral fitness, facilitating midge-to-midge transmission and subsequent replication, and further highlights the significance of *Culicoides* midges in VSV maintenance and transmission dynamics.

## 1. Introduction

Vesicular stomatitis virus (VSV; Rhabdoviridae) causes economically significant disease in cattle, horses, and swine; but can also infect sheep, goats, llamas, and alpacas. Clinical disease often presents as excessive salivation with vesicular lesions of the gums, tongue, naso-oral mucosa, teats, and coronary bands [1]. Because saliva and vesicular fluid can contain high titers of virus, VSV can be transmitted very efficiently between animals by direct contact, aerosol, and fomites. Additionally, VSV is vector-borne, with insects acting as mechanical and biological vectors [2]. Typically, VS occurs annually in enzootic regions of Central and South America, infecting a large percentage of susceptible species [1]. In contrast, in the U.S., it appears as sporadic epizootic incursions of viruses originating from enzootic regions in Mexico [3,4,5], resulting in single year or multi-year outbreaks with overwintering genotypes.

In the laboratory, VSV can rapidly replicate to high titers (8–10 Log_10_ plaque forming units (PFU)/mL in 24–48 h) in a variety of vertebrate and invertebrate host cells [6,7,8]. This remarkable plasticity has allowed the study of continuous replication in specific mammalian and insect cells in alternating environments [9,10,11]. In vitro, VSV displays high adaptability and elevated variation in fitness, sometimes accompanied by lower rates of nucleotide changes [6,10]. During replication events in single-host cell lines, VSV rapidly increases fitness to the cell line in use while decreasing replicative fitness to the bypassed host [9,12]. In nature, VSV remains relatively stable, with evolutionary patterns defined by similar ecological conditions rather than geographical origin or immunological selection [3,4]. Differences in post-translational glycosylation of the two potential N-linked glycosylation sites on the G protein in mammalian cells compared to insect cells is unknown. However, host cell enzymes are responsible for the addition of carbohydrates [9,10]; thus, the composition and degree of final sialylation is host-dependent. In addition to potential post-translational modification differences in host cells, the error-prone nature of VSV polymerase averages one mutation per genomic replication event, generating a wide diversity of genetic variants and quasispecies populations [3,6,9,10,13,14]. Viruses that experience spatial heterogeneity with lifecycles that include both mammalian and insect hosts likely have even greater genetic diversity for evolving fitness [15].

*Culicoides* biting midges (Diptera: Ceratopogonidae) are one of the primary vectors playing a key role in VSV transmission during outbreaks in the U.S. [1,3,16,17,18,19,20,21,22,23]. As characteristic of pool-feeders, *Culicoides* midges use their mouthparts to slash the animal’s epidermis, often in areas with little hair such as the naso-oral regions. Blood is ingested along with potential skin surface contaminants that pool in the wound. Because of this feeding mechanism, and because demonstrable viremia in infected animals is elusive [2,24], acquisition of VSV via blood feeding has been primarily considered to be dependent upon the availability of clinically infected hosts exhibiting excessive salivation and naso-oral vesicular lesions as sources of significant amounts of virus on the skin [24,25,26]. Once VSV is ingested by *Culicoides*, a pantropic systemic infection results [18], which—despite relatively low whole-body virus titers (3.3–4.6 Log_10_ PFU/mL [27])—also allows for highly efficient venereal transmission between female and male midges [28]. This midge-to-midge VSV transmission has been proposed as a maintenance mechanism by which epizootic virus genotypes may overwinter, resulting in a multi-year outbreak [3,29].

To understand the high efficiency seen in midge venereal transmission, we hypothesized that VSV populations propagated within *Culicoides* have increased fitness for subsequent midge infections. Here, we investigate whether the originating host cell species affects VSV fitness for *Culicoides sonorensis* midge infection.

## 2. Materials and Methods

### 2.1. Virus Strains

Three VSV-New Jersey (VSV-NJ) virus strains were used for midge infections; one laboratory-adapted positive control strain and two more recent outbreak isolates [30]. Strain 82-34333 is a Colorado bovine isolate from the 1982 epizootic outbreak [22] that has been passed numerous times in both mammalian and insect cells. Strain rNJ0612NME6 is a highly virulent synthesized equine isolate from a 2012 epizootic outbreak in New Mexico [31]. Strain rNJ0806VCB is a synthesized bovine isolate from a 2006 enzootic outbreak in Vera Cruz, Mexico [31]. Both synthesized strains were produced in BHK-21 cells [30] and are courtesy of S. Pauszek and L. Rodriguez, Plum Island Animal Disease Center, NY [31]. Virus strains were passaged one time at an MOI of 0.1 in porcine skin fibroblast cells (mammalian-derived VSV) and in *Culicoides* cells (insect-derived VSV) to produce high titer viral stocks that were stored at −80 °C for subsequent midge infections.

### 2.2. Cells

Porcine skin fibroblast cells (AG08113; Coriell Institute, Camden, NJ, USA) were maintained in Eagles MEM with Earle’s salts (Sigma, St. Louis, MO, USA) containing 2% FBS and 100 U penicillin/streptomycin sulfate at 37 °C with 5% CO_2_. *Culicoides* W8 cells [32] (USDA, Arthropod-Borne Animal Diseases Research Unit, Manhattan, KS, USA) were maintained in serum-free insect media (SFM) (24.5 g/L) supplemented with 0.4 g/L sodium bicarbonate, 0.0585 g/L l-glutamine, 0.006 g/L reduced glutathione, 0.03 g/L l-asparagine, 18 μL of 10 mg/L bovine insulin, and 5% FBS at 28 °C with a CO_2_ concentration of 0.2%. Vero MARU cells (VM; Middle America Research Unit, Panama City, Panama) grown in 199E media containing 2% FBS, 100 μg/mL of streptomycin, 100 units/mL penicillin, and 0.25 ug/mL of amphotericin B at 37 °C with 5% CO_2_ were used for detecting and titering infectious viruses from midge samples as described below.

### 2.3. VSV Infection of Culicoides Midges

Adult *Culicoides sonorensis* (Wirth and Jones) midges [33] (USDA, Arthropod-Borne Animal Diseases Research Unit, Manhattan, KS, USA) were used for all experiments. For viral exposure that bypassed the midge midgut barriers, females (1–3 days post-emergence) were anesthetized with CO_2_ and intrathoracically injected with 60 nL of each virus as previously described [28]. For all injections, a starting titer of 6.4 log_10_ PFU/mL was used for each strain; therefore, each midge received 2.1 log_10_ PFU.

For oral exposure, females (1–3 days post-emergence) were allowed to feed for 1 h on VSV-blood meals consisting of defibrinated sheep blood (Lampire Biological Products, Pipersville, PA, USA) mixed with each viral stock. To obtain the highest possible infection rates while reflecting the difference in viral titers observed in mammalian host vesicular lesions (up to 9 log_10_ PFU/mL fluid) compared to insect vectors (up to 5 log_10_ GE in whole bodies [27]), we used the highest average titer obtainable for each isolate from each cell source (Figure S1). Infectious blood meals with the virus isolates propagated in mammalian cells and insect cells had titers of 8.2 log_10_ PFU/mL and 6.4 log_10_ PFU/mL, respectively. After blood-feeding, fully engorged females were sorted from the unfed and partially fed and placed in cardboard maintenance cages. Fed and injected midge cages were maintained in environmental chambers at 25 ± 1 °C and 70–80% relative humidity with a 13:11 light: dark cycle and offered 10% sucrose solution ad libitum.

### 2.4. Sample Collection

To determine whole-body infection rates and titers of injected midges with each VSV isolate propagated in both cell types, midges were collected at 3- and 10-days post-injection (dpi). Whole midges were placed in either 300 µL of TRIzol (Invitrogen; Thermo Fisher Scientific, Inc., Waltham, MA, USA) for RNA isolation (*n* = 15) or 500 µL of antibiotic medium (199E cell culture medium containing 2% FBS and 400 U/mL penicillin, 400 μg/mL streptomycin, 200 μg/mL gentamycin, 5 μg/mL ciprofloxacin, 5 μg/mL amphotericin B) for virus isolation (*n* = 15), and stored at −80 °C until processed. To determine midgut infection and dissemination rates and associated viral titers of orally fed midges, heads (with salivary glands) were separated from bodies of midges fed with each isolate of both cell types. Samples were collected at 3-, 7-, and 10-days post-feeding (dpf) and placed in 300 µL of TRIzol for RNA isolation (*n* = 15) or 500 µL of antibiotic medium for virus isolation (*n* = 15) and stored at −80 °C until further processing. In addition, time-zero whole-body titer samples for both intrathoracically injected and orally fed midges of each group were collected in 300 µL of TRIzol (*n* = 5) or 500 µL of antibiotic medium (*n* = 5) immediately after infection.

### 2.5. RNA Extraction and RT-qPCR for Detection of VSV

Frozen samples in TRIzol were thawed on ice, homogenized with bead-beating, and total RNA was extracted using TRIzol-BCP (1-bromo-3chloropropane; Thermo Fisher Life Technologies, Waltham, MA, USA) as previously described [28]. RNA extracts were analyzed using TaqMan Fast Virus 1-Step Master Mix (Applied Biosystems; Thermo Fisher Scientific, Inc.) in a reverse transcriptase quantitative PCR (RT-qPCR) assay detecting VSV-NJ L (polymerase) gene as previously described [28]. Standard curves and cycle threshold (Ct) values were calculated using 7500 Fast Dx software v1.4 (Applied Biosystems; Thermo Fisher Scientific, Inc.). RT-qPCR reactions with Ct ≤ 36.5 were considered positive for VSV RNA [27]. Ct values plotted against the log of ssRNA VSV concentration and the linear regression (*y*= –3.30578*x* + 11.02683) were used to determine viral genomic equivalents per midge [28].

### 2.6. Virus Isolation from Infected Midges

Frozen midge samples stored in antibiotic media were thawed and homogenized as above. Samples were centrifuged at 12,000× *g* for 6 min to pellet debris and 200 μL of cleared supernatant was added to Vero monolayers with 85–90% confluency in 24-well plates. Additional media (300 μL) was added to each well, and plates were incubated at 37 °C for up to five days. Cytopathic effects (CPE) observed after two passages were used to indicate the presence or absence of infectious virus. Samples with positive CPE at the first passage were titered by plaque assay using 200 μL of the remaining original, non-passaged homogenate. Samples that showed CPE only after a second passage were below the limit of detection to quantify by plaque assay. Therefore, RT-qPCR testing of randomly selected wells was used to confirm that VSV was the source of the CPE observed. For plaque assays of original homogenates, 10-fold dilutions of cleared homogenate supernatants were used to inoculate (200 μL) monolayers of Vero cells at 90–95% confluency in 6-well plates. After a 1 h adsorption, each well was aspirated and monolayers were overlaid with 2% methylcellulose (M0512, Sigma, St. Louis, MO) mixed 1:1 with 2X 199E media. Plates were incubated for three days at 37 °C with 5% CO_2_ and stained with a crystal violet fixative stain (25% formaldehyde, 10% ethanol, 5% acetic acid, 1% crystal violet). Titers were reported as log_10_ PFU/mL of the original homogenate.

### 2.7. Statistical Analysis

Data from each virus from the same host species cell line were grouped for all analyses. Infection rates were calculated as the proportion of VSV-positive bodies, and dissemination rates were calculated as the proportion of VSV-positive heads. Two-way analysis of variance (ANOVA) with multiple comparisons was used to compare Ct values, infection, and dissemination rates. GraphPad Prism version 10 (GraphPad Software Inc., San Diego, CA, USA) was used for statistical analysis and the creation of graphs.

### 2.8. Virus Sequencing

NextSeq Illumina sequencing was performed by the Kansas State University Veterinary Diagnostic Laboratory. Consensus sequence identity was used to identify nucleotide changes between the original BHK low-passaged rNJ0612NME6 and rNJ0806VCB strains and these strains after one passage in the mammalian and insect cell lines. Sequences for the 82-34333 positive control reference isolate were excluded from the sequence analysis due to the lab-adapted nature of this isolate, which includes numerous passages in various mammalian and insect cell lines.

## 3. Results

### 3.1. Culicoides Midge Intrathoracic Infections

**Figure 1:**
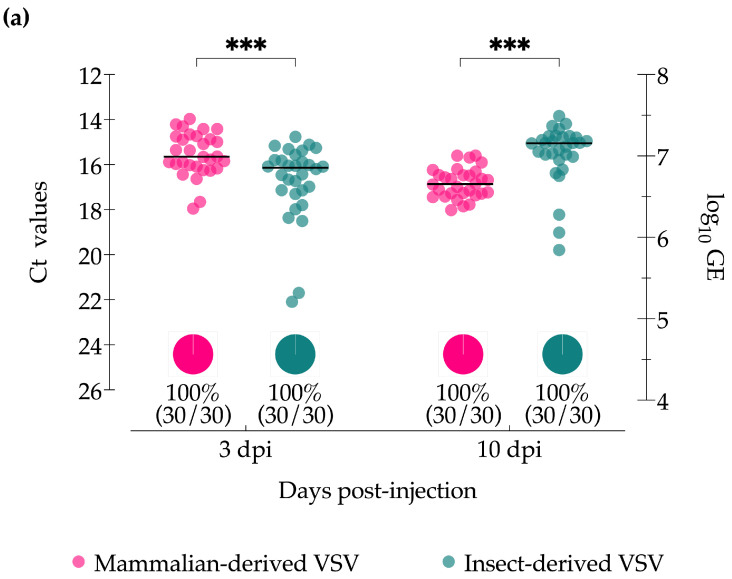

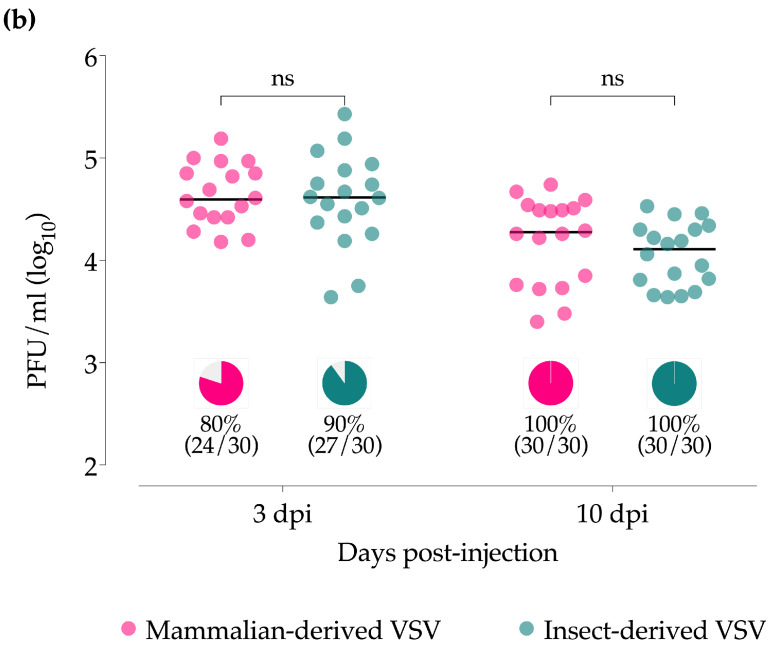
Virus detection in *Culicoides* midges intrathoracically injected with VSV-NJ propagated in either porcine skin fibroblast cells (pink) or *Culicoides* W8 cells (teal). (**a**) VSV titers of whole bodies of individual midges based on cycle threshold (Ct; left Y-axis) and viral genome equivalents (GE; right Y-axis) as detected by RT-qPCR. The percentage of VSV-positive midges detected by RT-qPCR (Ct ≤ 36) is represented by pie charts below each data set. (**b**) VSV infectious virus titer from whole bodies of individual midges as determined by plaque assay. The percentage of midges with infectious virus, as detected by initial CPE screening, is represented by pie charts below each data set. Two-way ANOVA with multiple comparisons was used to determine statistical significance as indicated (*p* > 0.05, ns, not significant; *** *p* < 0.001).

Immediately after injections (time zero), individual midge titers were below the limit of detection to quantify by plaque assay; however, all homogenates were positive by CPE screening after one or two passages (100%; 15/15), and detectable by RT-qPCR with genome equivalent (GE) levels ranging from 2.1–3.2 log_10_ virus particles (Figure S1), indicating all midges received statistically similar doses. Viral RNA was detected in all females, independent of cell source or sampling time point. Mammalian-derived VSV peaked earlier than insect-derived VSV with significantly higher virus titers (GE, as detected by RT-qPCR) at 3 days post-injection (dpi) (*p* = 0.0007; Figure 1a). Conversely, at 10 dpi, RNA was significantly higher in midges injected with insect-derived VSV (*p* = 0.0002; Figure 1a).

No significant differences in infection rates, as detected by CPE screening for infectious virus, were found in midges injected with either mammalian- or insect-derived VSV at either 3 dpi (80% and 90%) or 10 dpi (100% and 100%) (Figure 1b, pie charts). Nor were there significant differences in the average whole-body titers by plaque assaying the original homogenate from 3 dpi (4.5 and 4.6 log_10_ PFU/mL) or 10 dpi (4.2 and 4.1 log_10_ PFU/mL) (Figure 1b, scatter plots).

### 3.2. Culicoides Midge Oral Infections

Immediately after blood-feeding, ingested virus titers were measured in individual fully engorged females by plaque assay and RT-qPCR. As expected, mean titers of ingested virus, as detected by RT-qPCR, were higher in midges that fed on the higher titer mammalian-VSV blood meal (Ct mean 19.7, 5.5 log_10_ GE) than in midges that fed on the lower titer insect-VSV blood meal (Ct mean 26.8, 3.5 log_10_ GE) (Figure S1). Infectious virus titers in single whole bodies immediately after ingestion, as detected by plaque assay, were achieved in 100% (15/15) of the females fed with the mammalian-VSV, with a mean titer of 5 log_10_ PFU/mL; and in 66.7% (10/15) of the females fed with insect-VSV, with a mean titer of 1.9 log_10_ PFU/mL.

**Figure 2:**
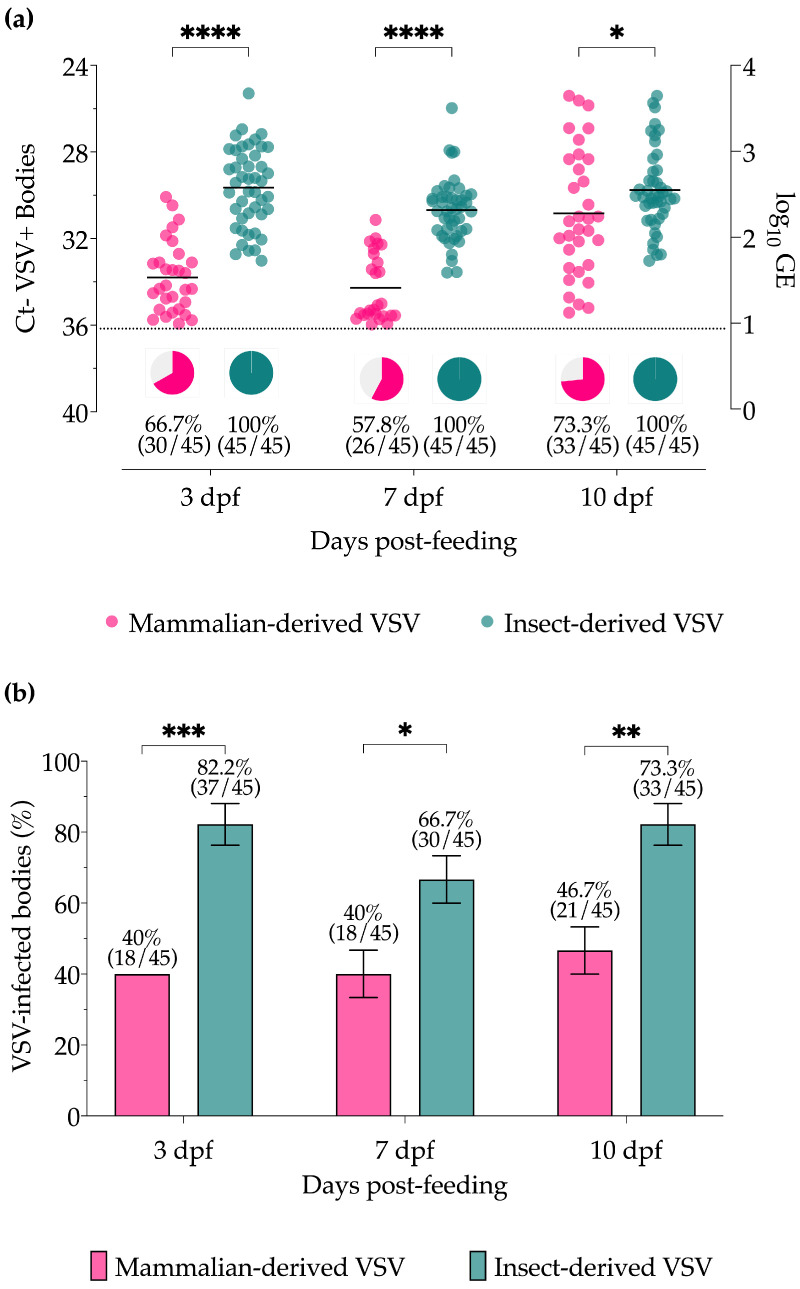
Virus detection in *Culicoides* midge bodies orally infected with VSV-NJ propagated in porcine skin fibroblast cells (pink) and *Culicoides* W8 cells (teal). (**a**) VSV titers of individual whole bodies (0 dpf) or decapitated bodies (3, 7, and 10 dpf) based on cycle threshold (Ct; left Y-axis) and viral genome equivalents (GE; right Y-axis) as detected by RT-qPCR. The percentage of positive midge bodies (Ct ≤ 36, dotted line) is represented by pie charts below each data set. (**b**) Proportional infection rates based on infectious virus detected by cytopathic effect (CPE) screening. Two-way ANOVA with multiple comparisons was used to determine statistical significance as indicated (*p* > 0.05, ns, not significant; * *p* ≤ 0.05; ** *p* < 0.01; *** *p* < 0.001; **** *p* ≤ 0.0001). Error bars represent the standard error of the mean (SEM).

At 3, 7, and 10 days post-feeding (dpf), individual decapitated bodies and their associated head/gland samples were assayed separately to determine VSV infection and dissemination rates, respectively. Although the highest infectious dose possible for the insect-VSV blood meals (6.4 log_10_ PFU/mL) was significantly lower than that of the mammalian-VSV blood meals (8.2 log_10_ PFU/mL), viral titers (GE) and the proportion of VSV RNA-positive bodies detected by RT-qPCR (Figure 2a) were significantly higher at all timepoints in midges fed with insect-derived blood meals (3 and 7 dpf *p* < 0.0001, 10 dpf *p* = 0.0436). Likewise, the percentages of VSV-positive bodies with infectious virus as detected by CPE (Figure 2b) were significantly higher at all time points in midges fed with insect-derived blood meals (3 dpf *p* = 0.0007, 7 dpf *p* = 0.0210, 10 dpf *p* = 0.0029). All body samples were below the detection limit of plaque assay for titration purposes.

**Figure 3:**
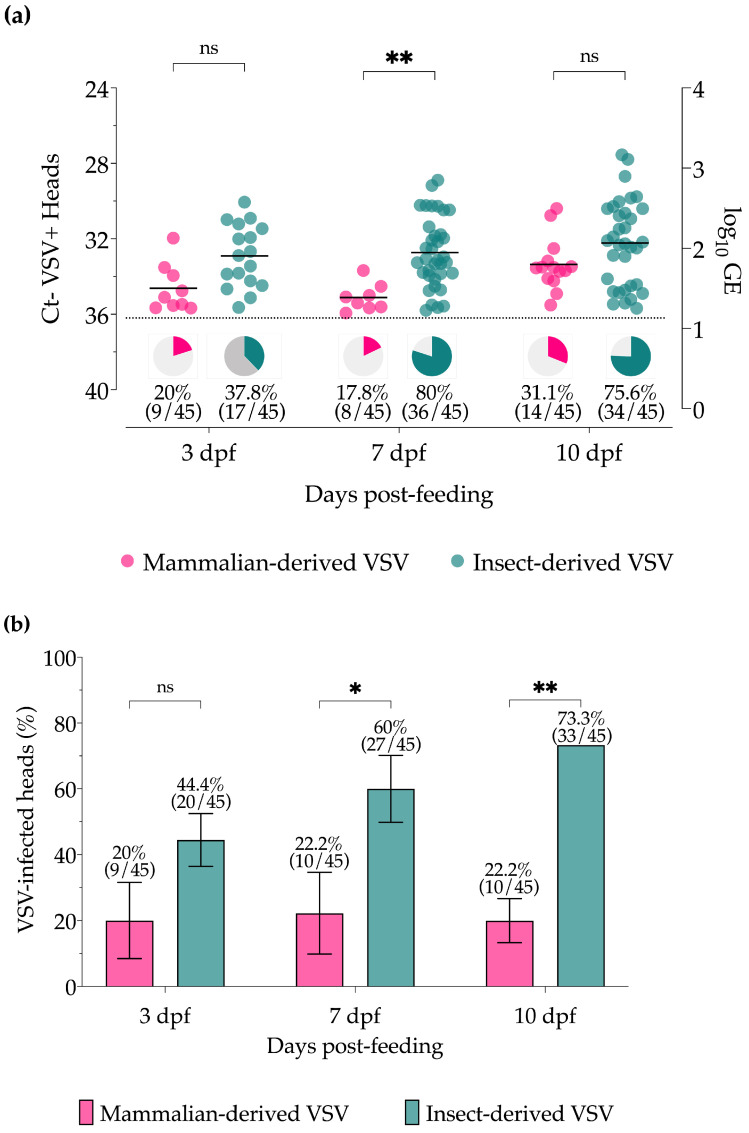
Virus detection in individual heads with glands of *Culicoides* midges orally infected with VSV-NJ propagated in porcine skin fibroblast cells (pink) and *Culicoides* W8 cells (teal). (**a**) VSV titers based on cycle threshold (Ct; left Y-axis) and viral genome equivalents (GE; right Y-axis) as detected by RT-qPCR. The percentage of positive midge heads (Ct ≤ 36, dotted line) is represented by pie charts below each data set. (**b**) Proportional infection rates based on infectious virus detected by cytopathic effect (CPE) screening. Two-way ANOVA with multiple comparisons was used to determine statistical significance as indicated (*p* > 0.05, ns, not significant; * *p* ≤ 0.05; ** *p* < 0.01). Error bars represent the standard error of the mean (SEM).

Viral titers (GE) and proportions of positive midge head/gland samples, as detected by RT-qPCR (Figure 3a), were also higher for insect-derived VSV blood-fed midges compared to mammalian-derived VSV blood-fed midges, although differences were only significant at 7 dpf (*p* = 0.0356). Likewise, the percentages of VSV-positive head/gland samples with infectious virus, as detected by CPE (Figure 3b), were higher at all time points for midges fed with insect-derived VSV blood meals with significant differences at 7 dpf (*p* = 0.0375) and 10 dpf (*p* = 0.0041). All head/gland samples were below the detection limit of the plaque assay for titration purposes.

### 3.3. Virus Sequences

The field isolate viral stocks rNJ0806VCB and rNJ0612NME6 (originally produced in BHK-21 cells) were replicated once in each of our test host cell sources (porcine and *Culicoides*) [30]. The laboratory stock isolate, 82-34333, had undergone multiple passages in multiple species of cell lines over 30 years. Since laboratory adaptation constitutes higher adaptability and variation in fitness due to nucleotide changes [6,10], we chose to compare consensus sequences of the more recent, low passage field isolates, rNJ0612NME6 and rNJ0806VCB, before and after the single passage in either mammalian (porcine skin fibroblast) or insect (*Culicoides* W8) cells to detect genome changes that might inform fitness differences. We found no nucleotide sequence changes. The sequence identity for both isolates in both cell lines had 100% identity to their BHK-parental strains.

## 4. Discussion

VSV epidemiology is very complex, involving a broad mammalian and insect host range with direct contact transmission between clinical mammalian hosts and vector-borne transmission via multiple vector species [2]. VS outbreaks in the U.S. can occur as single-year events. However, for the past 40 years, outbreak viruses have often overwintered between vector seasons, in the absence of infected animals, with the same viral genotype re-emerging in a second-year outbreak (1982–1983, 1985–1986, 1998–1998, 2004–2006, 2014–2015, 2019–2020) [3,16,34]. It has been suggested that VSV is being inter-epizootically maintained in yet-to-be-identified natural mammalian reservoirs [35,36], or within vector populations independent of feeding on viremic animals [28,37,38,39,40].

The VSV multi-host lifecycle demonstrates the flexibility of VSV populations and their ability to quickly adapt to infect and replicate in very different host cell environments. As in other arbovirus systems, alternating host transmission cycles may constrain the evolutionary rates through viral genetic stability without affecting viral fitness for either host alone [41,42,43,44]. Likewise, laboratory experiments have shown that despite VSV mutating approximately four times slower in insect cells [45], there are no effects in the number of mutations accumulated or any fitness increase or cost when there are alternating host passages [10,12]. However, host-derived non-genetic differences such as envelope composition and protein glycosylation can result due to substantial differences in post-translational machinery and the cell membrane constitution of insect and mammalian cells [46,47,48].

We hypothesized that VSV propagated within *Culicoides* may evolve increased fitness for insect cells, allowing persistence without deleterious effects to the population. This adaptability would further enable the re-emergence of an overwintered genotype when conditions for vectored bite transmission are once again ideal. For some arboviruses, alternate replication in two divergent hosts may reduce overall virus fitness in both hosts compared to a single-host virus lifecycle [49]. However, VSV populations can successfully replicate in multiple cellular environments without genetic fitness constraints [9,10,50]. Non-genetic differences accompanied by different replicative strategies in invertebrate vectors (persistent, non-lytic replication) and vertebrate hosts (acute, lytic infection) may play a significant role in shaping VSV evolution and therefore influence disease transmission dynamics. To evaluate the potential host-specific fitness increase, we evaluated the infection, replication, and dissemination differences of insect- and mammalian-derived viruses in a key VSV vector, *Culicoides sonorensis*.

First, we evaluated the viral infection dynamics after intrathoracic inoculation and after oral infection with VSV propagated in a mammalian host (porcine epidermal) and insect vector (*Culicoides* W8) cell lines. The results of independent infections with three different viral isolates were grouped by cell line to estimate the effects of the species-specific cell environment. In addition to highly sensitive molecular methods used to estimate viral load, infectious virus isolations by CPE and plaque assays represent a more relevant measurement of any infectiousness differences from host-derived factors. Disseminated virus in heads correlate with salivary gland infection and transmission potential [18]; therefore, the dissemination rates observed in this study may also indicate the magnitude of potential bite transmission competence by vectors infected with mammalian-sourced VSV versus insect-sourced VSV.

For many vector-borne diseases, most predictions often assume that vector infection rates are only driven by the magnitude of pathogen exposure [27,51,52,53]. However, the results of this study demonstrate that infection rates can also be strongly regulated by factors beyond the initial viral load. Here we have shown that both mammalian- and insect-derived viruses can efficiently infect midges and replicate equally well when the midgut barrier is circumvented via injection (Figure 1). However, with oral infection, where the virus was required to infect and escape the midgut to disseminate, insect-derived VSV showed significantly increased infection rates and titers in bodies, as well as infection and dissemination rates in heads/glands compared to midges infected with mammalian-derived VSV (Figure 2 and Figure 3) at all time points tested.

In midgut bypass trials, although all midges were injected with similar titers, higher mean titers of viral RNA were detected in midges injected with mammalian-derived VSV at the early time point (3 dpi). This may have triggered an enhanced immune response against these early VSV populations, resulting in the lower titer seen by day 10. Similar immune responses to mammalian-derived VSV may have influenced infection outcomes seen in oral exposures due to the higher starting virus titers delivered with the blood meal. However, increased fitness of the insect-derived virus at the midgut level (measured as increased genome replication) may also be explained by facilitation in the attachment and entry to the midgut epithelial cells due to enhanced insect-derived viral glycoprotein fusion with target cells [54,55,56], or even due to better immune evasion [57].

As similarly seen in field-collected insect and animal samples during outbreaks, it is important to note that during propagation of all the VSV-NJ isolates used, viral production (titer) was lower in the *Culicoides* cells than in the porcine cells (Figure S2). In insect cells, where some receptors may be present at lower densities than in mammalian cells, arbovirus selection may favor rapid entry over the ability to grow to high titers [44].

Within-host fitness experiments have been frequently conducted in cell culture experiments [9,10,50,54,58,59,60]; however, evaluating the impact of within-host fitness at the arthropod level can be more challenging to elucidate due to non-genetic differences between viruses derived from each host [46], or because the specific host environment may apply selection pressures to virus populations [61]. We believe that host-dependent modification differences in starting virus populations may dictate infection and replication success and subsequently influence transmission rates. Such differences may relate to the fitness and robustness of quasispecies populations derived from cells of the different species. As is indicated by the multi-host, multi-vector nature of VSV, it can infect multiple host cell types. In mammalian infections, VSV is restricted to skin lesions and their draining lymph nodes [62]. The representative mammalian cell line used in this study was similarly restrictive in that it was a clonal porcine skin fibroblast line. In contrast, VSV infection in midge vectors is pantropic, with virus detected in all cell types of all tissues [18]. Likewise, the representative insect cell line used in this study was similarly diverse in that it was made from whole midge embryos [32]. The spatial heterogeneity of such a polytypic cell line may result in a larger number of minority variants [15] which have a greater genetic diversity for evolving fitness more rapidly [63]. A more diverse virus population produced in midge cells may have an increased ability to rapidly mutate toward fitness and may help explain the efficiency seen in venereal transmission. An initial analysis of minority variants from raw genome sequence reads showed a trend toward increased diversity for virus propagated in *Culicoides* cells compared to the porcine cells, but was not conclusive as deeper reads are needed.

With dominant consensus viral genome sequences remaining the same, independent of the host cell environment, differences in observed fitness cannot be attributed to stabilized changes in the genome or protein-coding sequences, and may suggest that post-translational modifications contribute to fitness. In the case of enveloped viruses, host-encoded proteins are embedded in the host-derived lipid bilayer membrane envelope [64]. Alternating host variation in the lipid bilayer content has shown to have subsequent effects on Sindbis virus stability [59], and Ross River and Venezuelan equine encephalitis virus capacity to induce immune responses [57]. Moreover, additional host cytoplasmic factors can be incorporated into viral progeny [64]. Previous VSV proteomic analyses have shown host cell-type-dependent profiles influencing the infectivity of the resulting virions [64,65]. Additionally, the envelope of VSV is spiked with glycoprotein trimers (G protein), which are essential for cell receptor binding and entry, thereby playing a role in host range determination [66]. Host-dependent expression of multiple enzymes of the glycosylation pathways often results in the production of less complex glycans with high mannose content in insect cells [47,48]. Host-derived arboviral fitness due to glycosylation differences has been previously detected in Dengue [54], La Crosse [55], West Nile, and Sindbis viruses [56]. Likewise, host-dependent modifications of the VSV G protein [67,68] may constrain virion infectivity, cell tropism, and levels of virus replication in different host cell lines [65,66]. These mechanisms considered, effects seen from differences in the virion lipid bilayer envelope—and/or G protein glycosylation with mammalian-derived VSV—would be transient, as all progeny virions would have insect-derived envelopes and insect cell-dependent G protein glycosylation after one round of replication. In these studies, the observed fitness increase of insect-derived VSV was not transient; it was seen at all time points (3, 7, and 10 days) post-exposure.

Different selective pressures on arboviruses primarily transmitted through blood-feeding, versus transovarially or venereally, may also influence the degree of fitness observed at the vector level [41]. For viruses being maintained within vector populations, a stronger selective pressure for attenuation of within-host virulence accompanied by increased vector infectivity is expected [41,69]. VSV exhibits titer loss during persistent passages in insect cells [50] and after several generations of solely transovarial transmission within sand flies [38,39], indicating the requirement of clinically infected mammalian hosts to perpetuate the VSV lifecycle. Nevertheless, our results show that VSV propagation in *Culicoides* cells is significantly more fit for efficient replication within midges and for subsequent midge-to-midge transmission. Our results reinforce the idea that host–virus interactions play a critical role in VSV epidemiology and suggest that vector-mediated selection may play a role in virus persistence during interepizootic periods.

## Data Availability

The data presented in this study will be made available on Ag Data Commons and available by request from the authors.

## Supplementary Materials

The following supporting information can be downloaded at: https://www.mdpi.com/article/10.3390/insects15010034/s1, Figure S1: Individual midge titers at time zero; Figure S2: In vitro growth kinetics of VSV-NJ.
