# Supplementary material for: Culicoides-Specific Fitness Increase of Vesicular Stomatitis Virus in Insect-to-Insect Infections
Source: Insects. 2024 Jan 5;15(1):34. doi: 10.3390/insects15010034 (PMC10816812; doi:10.3390/insects15010034)
Supplement: Supplementary file 1 [file insects-15-00034-s001.zip › insects-2789991-supplementary.pdf]

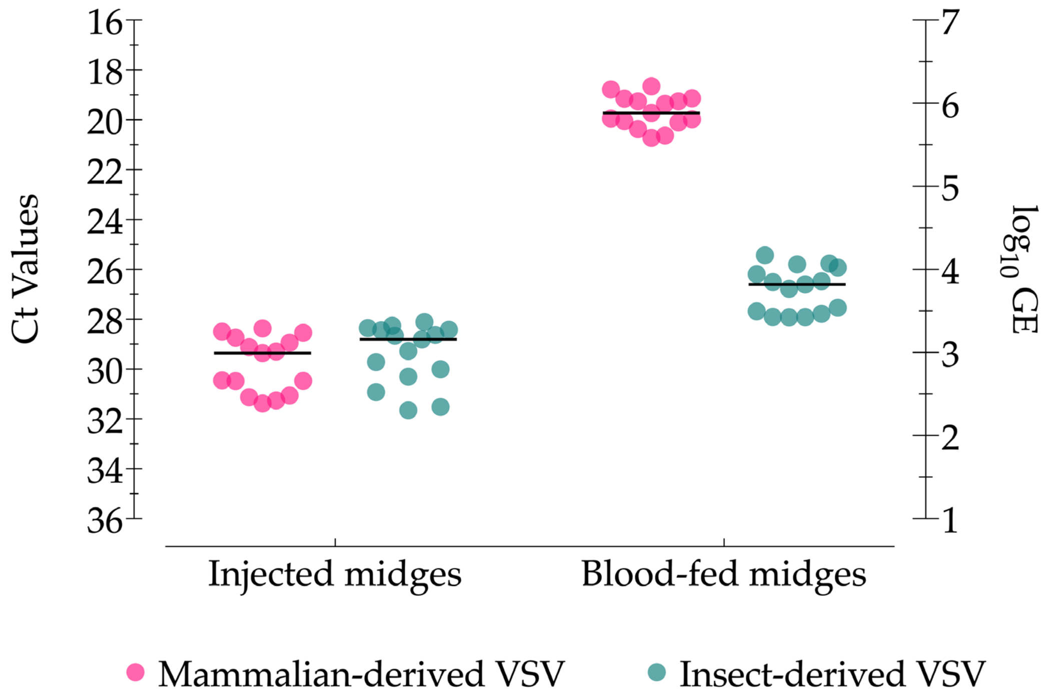

Figure S1. Individual midge titers at time zero. VSV titers of individual whole midge bodies collected (**a**) immediately after injections and immediately after blood-feeding as detected by RT-qPCR. Cycle threshold (Ct; left *Y*-axis) and viral genome equivalents (GE; right *Y*-axis).

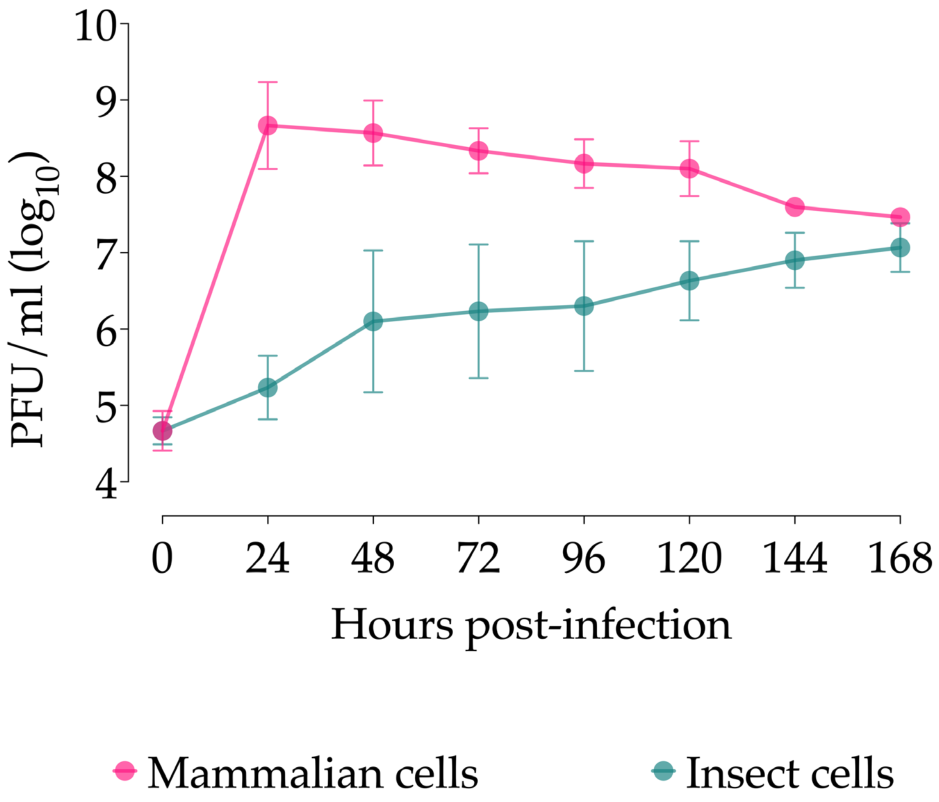

Figure S2. *In vitro* growth kinetics of VSV-NJ. Isolates 82-34333r, NJ0806VCB, and rNJ0612NME6 propagated in mammalian cell culture (porcine skin fibroblast, pink) and insect cell culture (*Culicoides* W8, teal). All cell lines were infected at an MOI of 0.1 and harvested at indicated time points. Infections were titrated in Vero cells. Error bars represent the standard error of the mean (SEM).
